# Editorial: CD4+ T cells in HIV: A Friend or a Foe?

**DOI:** 10.3389/fimmu.2023.1203531

**Published:** 2023-07-11

**Authors:** Sakthivel Govindaraj, Hemalatha Babu, Sunil Kannanganat, Monica Vaccari, Constantinos Petrovas, Vijayakumar Velu

**Affiliations:** ^1^ Department of Pathology and Laboratory Medicine, Emory University School of Medicine, Atlanta, GA, United States; ^2^ Division of Microbiology and Immunology, Emory Vaccine Center, Emory University, Atlanta, GA, United States; ^3^ Department of Pathology and Genomic Medicine, Houston Methodist Hospital Research Institute, Houston, TX, United States; ^4^ Division of Immunology, Tulane National Primate Research Center, Covington, LA, United States; ^5^ Department of Microbiology and Immunology, Tulane School of Medicine, New Orleans, LA, United States; ^6^ Department of Laboratory Medicine and Pathology, Institute of Pathology, Lausanne University Hospital, University of Lausanne, Lausanne, Switzerland

**Keywords:** HIV, CD4+ T cells, reservoirs, HIV cure, follicular T helper cells

Currently, there are approximately 38.4 million individuals living with the Human Immunodeficiency Virus (HIV), of which 36.7 million adults, 1.7 million children (<15 years old), with 54% of cases being females. Since the start of the HIV epidemic, an estimated 84.2 million individuals have been infected with the virus. Tragically, this global health crisis has resulted in the loss of approximately 40 million lives. In 2021 the World Health Organization (WHO) estimated 1.5 million new infections (1). In the early stages of HIV infection, several important events occur within CD4+ T cells, which are a primary target of the virus. CD4+ T cell depletion: HIV infects and destroys CD4+ T cells during the replication process. If left untreated, HIV is the virus kills infected cells directly and indirectly through immune responses that cause cell death. This progressive loss of CD4+ T cells weakens the immune system over time even following antiretroviral treatment (cART) in HIV infected individuals (2). HIV-infected individuals often experience imbalances in CD4+ T cell levels and function. While cART is highly effective in suppressing viral replication and restoring immune function, it may not completely normalize CD4+ T cell counts or fully restore immune balance in all individuals. Some HIV reservoirs, such as latently infected CD4+ T cells or tissues with lower drug penetration, may continue to harbor the virus, and chronic immune activation and inflammation associated with HIV infection can lead to immune exhaustion, where immune cells, including CD4+ T cells, become functionally impaired and less responsive. Despite extensive research, the mechanisms underlying CD4+ T cell loss and dysfunction in HIV infection are not fully understood (3). This edited topic is aimed at shedding lights on the effects of HIV infection on CD4+ T cells, considering their complexity in terms of heterogeneity and tissue distribution (4–7).

The relationship between HIV infection and CD4+ T cell population is intricate, partly because of their important functions and heterogenous nature. CD4^+^ T cell responses are central in orchestrating the adaptive immune responses to pathogens (8). Moreover, they are critical in inducing long-lasting vaccine-mediated protection (9). Their main function is to provide help adaptive immune cells; they support antibody production by B cells, affinity maturation, and the selection and they support the generation of long-lasting effective CD8+ T cells, and their antiviral functions. In the last decade, however, our understanding of CD4+ T cell immunology has grown, and it has become clear that CD4+ T cells function extends well beyond the simplistic view of B and T cell help. Many new CD4+ T cell subsets have been discovered (10) with highly specialized and distinct functions and tissue distribution; these subsets include negative regulators of the immune system, T regulatory cells (T-regs), preferentially mucosal CD4+ T cells Th17 and Th9 cells, and B zone resident T follicular helper (Tfh) cells and T follicular regulatory cells (11, 12). All subsets can be defined by specific surface and intracellular markers, including their own unique transcription factor and cytokine profiles. However, many questions remain elusive about their specific role in HIV infection. These subsets are not equally susceptible and each of them may play a unique yet significant role in establishing and maintaining virus reservoirs. Moreover, difference in HIV susceptibility of various CD4 T cells and the loss of CD4+ T cells very early in infection and the imbalance between these subsets is likely to affect the effectiveness of prophylactics vaccines and therapeutic approaches for HIV.

Activated CD4+ T cells located at mucosal sites are the main targets for HIV infection. Therefore, interventions aimed at reducing the vulnerability of these mucosal targets, particularly in the genital tract which serves as the portal of entry for HIV, could potentially lower the risk of HIV acquisition. Lajoie et al. investigated the effect of acetylsalicylic acid (ASA), a well-known and safe systemic anti-inflammatory drug, on T cell activation of the vaginal tract. The study was conducted in a cohort of HIV-uninfected women from Kenya who were given oral ASA at a low dose of 81mg daily for six weeks. Participants were followed for one month to establish a baseline immune activation in the blood and the female genital tract (FGT). Changes to T cell immune activation were measured both systemically and in the mucosal compartment, relative to baseline levels. The authors found that while concentration of ASA in the blood was 58% higher than the level measured in the FGT changes where noticeable in both sites. The blood level of ASA was correlated with lower levels of Th17 cells, CCR5 expressing target CD4+ T cells, and a specialized subset of CD8+ cells called Tc17. Importantly, in the FGT, low- dose ASA resulted in decreased levels of activated CD4+ T cells. Activation was measured by expression of the CCR5, CD95, and CD161+, all of which are markers associate with increased HIV replication (13, 14). This study suggests that ASA may be used to decrease activated FGT CD4+ T cells, thereby potentially reducing the risk of HIV vaginal transmission.

Recent studies showed that CD4+ T cells expressing the surface receptor glycoprotein CD32 are more susceptible to infection, and virological analyses revealed that CD32+ CD4+ T cells express high levels of inflammatory markers (HIV co-receptor CCR5, and PD-1, CXCR3) and confirming their heightened susceptibility to HIV infection and serve as a HIV reservoir. Overall, the study characterized a new susceptible CD4+ T cell subset in monkeys that exhibits an activated profile and higher expression of markers associated with HIV-infected and/or reservoir cells, and thus may represent a novel therapeutic target (Huot et al.).

Olwenyi et al. examined a subset of cytotoxic (CTLs) CD4+ T cells and investigated their contribution to HIV persistence. CD4+ CTLs T cells can reduce viral replication and have been shown to kill infected macrophages, by secreting granzyme B and perforin and to kill the target cells in an MHC class II-restricted fashion. While they may play important roles in antiviral immunity, the lack of robust markers in various animal models limits understanding of their role in HIV immunopathology. The group found that CD4+ T cells expressing high levels of the integrin beta CD29 are highly cytotoxic *in vitro*, and they corroborated the use of CD29 as a marker to detect CD4+ CTLs T cells *ex vivo*, using PBMCs obtained from SIV infected macaques. Interestingly, CD29+ CD4+ T cells are depleted during untreated SIV infection and reconstituted after early initiation of ART. Moreover, functional characterization of these cells showed that they produce IL-21 and granzyme B and that their ability to secrete anti-viral cytokines is reduced by morphine, an opioid drug. These findings are relevant because they suggest that CD4+ CTLs play a crucial role in limiting HIV pathogenesis and persistence (15).

Despite the remarkable effectiveness of combination antiretroviral therapy (cART) in suppressing HIV replication in the bloodstream over long periods of time, achieving sustained virologic remission in HIV-infected individuals continues to be a formidable challenge. One of the primary reasons for the challenge in achieving sustained virologic remission is the presence of latent HIV reservoirs. These reservoirs consist of HIV-infected cells that are in a dormant or inactive state, allowing them to evade the effects of antiretroviral therapy. While cART effectively suppresses active viral replication, it is unable to eliminate these latent reservoirs. As a result, even with long-term treatment, the potential for viral rebound and the need for ongoing therapy persist. Efforts to target and eliminate these latent reservoirs are a critical focus of HIV cure research. Several attempts have been made to clear viral reservoirs including cART (16), latency-reversing agents (LRA) (17), immune based therapies (15), gene editing and gene therapy (18), stem cell transplantation (19) and combination therapies (20). In the study by de Armas et al., FDA-approved JAK1/2 inhibitors, ruxolitinib, and baricitinib, were tested as a potential therapeutic strategy to target latent reservoirs. The study used the dual reporter virus HIVGKO to investigate latency establishment and maintenance in lymphoid-derived CD4+ T cells. Single-cell technologies were integrated to evaluate protein expression, host gene expression, and HIV transcript expression, and identify and analyze latently infected cells. The latency establishment and maintenance were validated using the tonsillar CD4+ T cells *in vitro* method. The results from this study show that CD4+ T cells with latent infection exhibit similar activation profiles as productively infected cells. Single-cell RNAseq analysis revealed that Sirtuin signaling, Oxidative Phosphorylation, Mitochondrial Dysfunction, and EIF2 Signaling were enriched when comparing latent cells versus productively infected cells. In addition, molecular JAK1/2 inhibition resulted in dose-dependent downregulation of activation markers on CD4+ T cells. Moreover, the expression of activation markers such as CD25 and CD69 was affected by both drugs. Finally, HIV reactivation above the level of spontaneous reactivation was observed in the presence of a LRA, but pretreatment of cells with baricitinib abrogated the response, with the greatest effect occurring at the highest concentration of the drug. It is therefore, plausible that JAK1/2 inhibition may block HIV-induced IFN and thus lead to a reduction in viral reservoirs.

The crucial involvement of T follicular helper (Tfh) cells in B cell responses, including against HIV is widely recognized (10, 21, 22). However, while harnessing Tfh may be desirable, they also harbor the virus (23). Moreover, there remains uncertainty regarding which specific subset of Tfh cells, either from lymph nodes or circulating in the blood, harbors a higher reservoir burden. This is due to the heterogeneity within the Tfh cell population, it is challenging to determine their unique and complex role in HIV pathogenesis and their potential in HIV prevention. Therefore, a better understanding of the mechanisms underlying Tfh cell differentiation and function in lymphoid tissues and immune contexts is essential for developing effective treatment strategies for HIV.

Moysi et al. conducted a study in which they developed and optimized eight multispectral confocal microscopy immunofluorescence panels for a comprehensive characterization of Tfh and other important cells (Tregs, CD8 and macrophages) using formalin-fixed paraffin embedded human lymphoid tissue samples. Here the authors discuss how the resulting multispectral confocal datasets can be analyzed quantitatively using a pipeline named HistoCytometry to gather information about relative frequencies and spatial distributions of immune cells. To characterize cells harboring the actively transcribed virus, the study used an *in-situ* hybridization assay in combination with additional protein markers (multispectral RNAscope). By increasing the dimensionality and predictive power of flow cytometry and single-cell RNA expression analyses. Applying this methodology to lymphoid tissues may provide an opportunity to investigate multiple immune cell targets of interest simultaneously, with improved resolution and reproducibility. This approach may therefore serve as a valuable tool to guide studies on CD4+ T-cells in the context of infection and vaccination, offering deeper insights into their dynamics and responses (24).

Studying HIV-2 in comparison to HIV-1 provides several important insights and contributions to our understanding of HIV infection disease progression and clinical outcomes: HIV-2 infection typically progresses more slowly compared to HIV-1, leading to a milder disease course. Thus, studying CD4 T cells in HIV-2 may allow to investigate factors related to this slower progression. Ponnan et al. evaluated the different subsets of CD4+ T cells, including Tfhs, in HIV-2 infected individuals to understand their role in controlling virus replication and delaying disease progression. The authors observed elevated levels of CD4+ Tfh and Stem cell memory Tscm CD4+ cells, as well as an abundance of memory and effector T cells in HIV-2-infected individuals. Additionally, they found increased frequencies of CXCR5+ CD8+ T cells and CD8+ Tscm cells, as well as memory B cells responsible for NAb development in HIV-2 infected persons. Interestingly, the frequency of memory CD4+ T cells and memory B cells significantly correlated with neutralizing antibody titers in HIV-2-infected individuals. Overall, this study suggests that a more robust CD4+ T cell response that supports B cell differentiation, antibody production, and CD8+ T cell development in HIV-2 infected individuals contributes to better control of the virus and slower disease progression.

Luo et al. conducted a study to explore the diversity of CD4+ T cell subsets in terms of their susceptibility or resistance to HIV-associated killing to understand the pathogenesis of disease progression in CD4+ T cells. The authors used lymphoid cells obtained from human tonsils and employed mass cytometry and single-cell RNA-seq techniques to test their hypothesis. Their results indicated that subsets of CD4+ T cells that are preferentially killed express CXCR4 and CXCR5, a marker used to identify Tfh and the receptor allowing CD4 T cells to migrate to the B cell zone. Further analysis using single-cell RNA-seq showed that the preferentially killed subsets express genes that favor abortive infection and pyroptosis. These findings highlight the complex interplay between HIV and distinct tissue based CD4+ T cell subsets, including Thfs. The study also found that bystander death is more likely to happen when infection involves X4-tropic viruses and is associated with higher expression levels of CXCR4. Additionally, susceptible bystander cells appear to be primed for death by pyroptosis, as indicated by increased mRNA expression of inflammatory caspases (caspase 1 and caspase 4) as well as the pyroptotic executioner, gasdermin D (25).

The review by Onabajo and Mattapallil discusses the effect of HIV infection on Tfh cells and mucosal antibody responses in the gastrointestinal tract (GIT). They highlighted the consequences of such immune deregulations on gut dysbiosis and pathogenesis during chronic HIV infection. Mucosal immunoglobulins A (IgA) play a critical role in protecting the GIT from invasive bacteria. The study also suggests that the dysregulation of Tfh cells leads to compromise B cell responses, especially the secretion of microbe-specific IgA, and it is likely to drive gut microbial dysbiosis during chronic HIV infection. Hence, preserving and maintaining Tfh cell responses in the mucosa could potentially restore high-affinity mucosal IgA, aiding in protecting the mucosal epithelial barrier from invasive dysbiotic bacteria (26).

T cell responses are intricately regulated by a complex network of non-coding RNAs, which includes microRNAs (miRNAs) and long non-coding RNAs (lncRNAs) (27, 28). These non-coding RNAs play crucial roles in modulating gene expression, influencing various aspects of T cell development, activation, differentiation, and effector functions. MicroRNAs are small RNA molecules that can bind to target messenger RNAs (mRNAs), leading to their degradation or inhibition of translation, thereby regulating the expression of specific genes. On the other hand, long non-coding RNAs are larger RNA molecules that can interact with chromatin, proteins, and other RNA molecules to regulate gene expression at multiple levels (24, 29, 30). Together, these non-coding RNAs contribute to the fine-tuning and coordination of T cell responses in both health and disease. Further research into the functions and mechanisms of non-coding RNAs in T cells continues to expand our understanding of immune regulation and may provide insights into HIV pathogenesis. The study conducted by Nguyen et al. aimed to investigate the role of long non-coding RNAs in the development of CD4+ T cell dysfunction and apoptosis in people living with HIV (PLHIV). The results revealed that the growth arrest-specific transcript 5 (GAS5) negatively regulates miR-21 expression, which controls critical signaling pathways involved in DNA damage and cellular response. Prolonged T cell stimulation reduced GAS5 and increased miR-21, leading to dysfunction and apoptosis in CD4+ T cells. These findings suggest that GAS5 regulates TCR-mediated activation and apoptosis in CD4 T cells during HIV infection through miR-21-mediated signaling pathway (37).

Van de Wijer et al. conducted a study aimed to investigate long-term changes in the immune system of people living with HIV (PLWH) who have successfully undergone treatment. Samples obtained from a cohort of 211 PLWH on stable antiretroviral therapy and 56 HIV-uninfected controls were assessed using flow cytometry to analyze 108 white blood cell (WBC) populations. The results indicated significant differences in T cell maturation and differentiation between PLWH and HIV-uninfected controls, with the PLWH exhibiting reduced percentages of CD4+ T cells and naive T cells, and increased percentages of CD8+ T cells, effector T cells, and T helper 17 (Th17) cells. In addition, the Th17/regulatory T cell (T-reg) ratios were found to be increased. PLWH also had altered B cell maturation, with reduced percentages of memory B cells and increased numbers of plasma blasts. Overall, this study suggests that the composition of circulating innate and adaptive immune cells is altered in PLHIV receiving cART for more than six months. The findings further suggest that while some adaptive immune responses such as Th17 are preserved, IFN-g responses are compromised in PLHIV (Van de Wijer et al.).

The manuscript by Zhu et al. conducted a study to investigate the immunological failure seen in immunological non-responders (InRs), HIV-infected individuals treated with ART, although successful in suppressing viral replication, they cannot properly reconstitute circulating CD4+ T-cell numbers to immunocompetent levels. The study found that InRs had platelets containing infectious HIV, which presence was linked to T-cell dysfunctions. The authors showed that platelet-T cell conjugates were more frequent among CD4+ T cells in InRs with HIV-containing platelets (<350 CD4+ T cells/ml blood for >1 year) compared to healthy donors or IRs (>350 CD4+ T cells/ml). Based on these findings, the authors suggest that targeted therapy aimed at platelets may improve immune reconstitution in InRs (39).

The systematic review by Le Hingrat et al. focused on the dynamics of CD4+ T cells in SIV and HIV. The reviewed literature includes studies on the possible mechanism of CD4+ T cell depletion and restoration in disease progression, and during early ART, respectively. The role of cell death and loss of CD4+ cell subsets in gut health are discussed. Reviewed studies suggest that the loss of CD4+ T cells contributes to mucosal inflammation and enteropathy, which weakens the mucosal barrier, leading to microbial translocation, a major driver of immune activation/inflammation. Studies also suggest that the loss of CD4+ T cells drives opportunistic infections, cancers, and comorbidities. Additionally, the authors discussed the crucial role of Th17 cells in maintaining gut integrity and protecting against bacterial and fungal infections (40).

In their systematic review, Inderbitzin et al. utilized primary CD4+ T cell models to investigate HIV-1 latency and potential cure strategies. The authors performed a pooled data analysis, comparing transcriptome profiles of latently and reactivated HIV-1 infected cells from five *in vitro* primary CD4+ T cell models and two *ex-vivo* studies of reactivated HIV-1 infected primary CD4+ T cells from HIV-1 infected individuals. The authors found that natural ligands CCL4, CCL5 and chemokine receptor CXCR6 were predominantly downregulated in latently infected cells, while genes associated with apoptosis, cell cycle, and HLA class II were upregulated in reactivated infected cells. Furthermore, they identified 5 genes FRY, GCSAM, GNLY, GPR15 and MSRB2 that co-occurred in both latently- and reactivated HIV-1 infected primary CD4+ T cells. The upregulation of MSRB2 in latently HIV-1 infected primary CD4+ T cells could inhibit apoptosis, while downregulation in reactivated HIV-1 infected genes leads to apoptosis due to the cytotoxic response of LRA or infectious virus particle release. This study sheds light on the differentially expressed genes that may contribute to the HIV-1 latency (41).

Both SARS-CoV-2 and HIV can dysregulate the immune system, leading to acute and long lasting inflammation, albeit through different mechanisms. By comparing the immune dysregulation caused by these viruses, we can identify commonalities and differences, which may inform the development of immunomodulatory therapies and approaches to mitigate immune-mediated damage in COVID-19. Peng et al. provided an overview of host characteristics and hyper-inflammatory responses in COVID-19 and HIV infection to understand the mechanisms of T-cell dysfunction better. The study summarized that both HIV-1 and SARS-CoV-2 infections are associated with CD4+ T cell loss and immunodeficiency. Direct attacks on CD4+ T cells, immune activation, and redistribution of CD4+ T cells contribute to CD4+ T cell lymphopenia in both diseases, but in different proportions. During the period of immunodeficiency, systemic inflammation may be fueled by a leaky gut and lead to severe complications. However, the co-occurrence of HIV and COVID-19 does not seem to increase the occurrence of COVID-19 or cause excess morbidity and mortality among people living with HIV with symptomatic COVID-19. Overall, the experience in HIV clinical management and past clinical trials represents a special use case for innovative studies aimed at increasing CD4+ T cell function and reducing COVID-19 severity (42).

The review by Gobran et al. discuss the current literature on the pathogenesis of HIV/HCV coinfection, the impact of HCV coinfection on HIV disease progression in the presence of ART, the effects of HIV on HCV-associated liver morbidity. Globally, approximately 2.3 million individuals are affected by a co-infection of the HIV and hepatitis C virus (HCV), with about 6.2% of people living with HIV (PLWH) also co-infected with HCV. PLWH have a six times higher incidence of being infected with HCV compared to those who are HIV-negative. The prevalence of co-infection is highest among individuals who inject drugs (PWID) and men who have sex with men (MSM). When HIV and HCV coexist, HCV’s natural history is negatively affected, leading to higher rates of HCV persistence following acute infection, higher viral loads, accelerated progression of liver fibrosis, and development of end-stage liver disease compared to HCV infection alone. Similarly, HCV coinfection harms the homeostasis of CD4+ T cell counts and facilitates HIV replication and viral reservoir persistence in PLWH receiving antiretroviral therapies (ART). Moreover, the consequences of Direct Acting antiviral (DAA)-mediated HCV cure on immune reconstitution and HIV reservoir persistence in coinfected patients is also discussed. The author comment on studies where it was shown that HIV reservoir is higher in HCV/HIV coinfected individuals than in those with HIV mono-infection, and DAA-mediated HCV cure does not reduce HIV persistence or protect against HCV reinfection. Therefore, developing effective treatment and prophylactic anti-HCV and anti-HIV vaccines remains a priority to achieve HCV/HIV elimination (43).

HIV vaccine and cure strategies aim to enhance immune responses to achieve long-lasting and, ideally, sterilizing immunity against the virus. The presence of antibodies (preferably broadly neutralizing) and cytotoxic T cells at the site of viral entry is crucial in stopping infection and virus dissemination. In contrast, CD4+ T cells are necessary for mounting a robust vaccine response. However, inducing a strong CD4+ T cell response in HIV infection is a double-edged sword. This is because CD4+ T cells also serve as primary targets of HIV infection and activated CD4+ T cells can be preferentially infected during the early stages of infection. High frequencies of vaccine-elicited CD4+ T cells may also limit vaccine-mediated protection. The specific lineages of CD4+ T cells have been reported to act as HIV reservoirs during chronic HIV infection (10, 25, 31). Furthermore, accumulating evidence suggests that some forms of vaccine specific CD4+ T cell responses may increase susceptibility to HIV infection.

While CD4 T cells are important to generate and maintain effective B and CD8 T cells responses, CD4 T cell induction trough vaccination may be a double edge sword. Evidence is mounting that some of vaccine induced CD4+ T cell responses may increase susceptibility to HIV infection. Results from the STEP HIV vaccine trial highlighted a potential role for total activated vaccine induced CD4+ T cells in promoting HIV acquisition (26, 32, 33). The trial also revealed that preexisting adenoviral immunity triggered a recall response, leading to the activation of adenovirus specific CD4+ T cells. These activated CD4+ T cells could potentially become additional target cells and may have increased the susceptibility to HIV infection in the vaccine arm of the study (34). Interestingly recent studies in macaques showed that the DNA prime/modified vaccinia Ankara boost vaccine induced interferon-γ (IFN-γ^+^) CD4+ T cells (Th1 cells), which rapidly migrate to multiple tissues including colon, cervix, and vaginal mucosa. These mucosal Th1 cells persisted at higher frequencies and expressed higher density of CCR5, a viral coreceptor, compared to cells in blood. After intravaginal or intrarectal simian immunodeficiency virus (SIV)/simian-human immunodeficiency virus (SHIV) challenges, strong vaccine protection was evident only in animals that had lower frequencies of vaccine-specific Th1 cells but not in animals that had higher frequencies of Th1 cells, despite comparable vaccine-induced humoral and CD8+ T cell immunity in both groups (35). These results corroborate the notion that high and persisting frequencies of HIV vaccine-induced Th1-biased CD4+ T cells in the intestinal and genital mucosa can mitigate beneficial effects of protective antibodies and CD8+ T cells, highlighting the critical need to better understand how to harness protective responses without increasing HIV susceptibility (36). Given the paradoxical role of CD4+ T cells in HIV infection, understanding the interplay between vaccine-induced CD4+ T cells and vaccine-mediated protection is paramount. To tip the balance in favor of vaccine-mediated protection, a rational design and delivery of vaccines in combination with newer immunotherapeutic strategies like engineered T cells (such as CAR/TCR-T cells) are required. This Research Topic is expected to bring together the current knowledge of CD4+ T cells as important central mediators of adaptive immunity while also being the cellular reservoir and targets for the multiplication and persistence of HIV. Recent research has provided valuable insights into the mechanisms underlying CD4+ T cell depletion and dysfunction in HIV infection, as well as the factors contributing to the natural control of the virus. This Research Topic presents a comprehensive overview (Highlights summarized in **Table 1**) of these recent advances, focusing on CD4+ T cell dynamics in HIV immunology and pathogenesis. By summarizing the latest findings and highlighting key knowledge gaps, this Research Topic aims to provide a useful resource for researchers and clinicians working on HIV/AIDS, as well as to inspire further research into the discovery of novel therapeutic targets.

**Table 1:** Highlights of the articles presented in the Research Topic *CD4+ T cells in HIV: A Friend or a Foe*?

Article Description	Citation
Acetylsalicylic acid (ASA) use was associated with a decrease in activated CD4+ T cells CD4+CCR5+CD161+ and CD4+CCR5+CD95+ cells, both at the systemic and female genital tract.	Lajoie et al.
The CXCR5+ and CXCR4+ CD4 T cell subsets are preferentially killed during HIV infection, while those that are preferentially infected exhibit an activated and exhausted effector memory cell phenotype. Single-cell RNA-seq analysis showed that the preferentially killed subsets express genes that favor abortive infection and pyroptosis, and exhibits increased mRNA expression of inflammatory caspases (caspase 1 and caspase 4) as well as the pyroptotic executioner, gasdermin D.	Luo et al.
The CD32+CD4+ T cells exhibit an activated profile and higher expression of markers associated with HIV-infected and/or reservoir cells.	Huot et al.
The CD29hi CD4 T cells can serve as a reliable marker to identify CD4+ cytotoxic T lymphocytes (CTLs) in rhesus macaques. These CTLs secrete higher levels of cytotoxic and proinflammatory cytokines, and elevated expression of either IL-21 or granzyme B hi T Bet+ upon antigen stimulation. CD4+ CTLs play a crucial role in limiting SIV pathogenesis and persistence. However, their expression is reduced following morphine exposure.	Olwenyi et al.
JAK1/2 inhibition by baricitinib may block HIV-induced IFN and lead to a reduction in the HIV reservoir.	de Armas et al.
This study optimized eight multispectral confocal microscopy immunofluorescence panels for a comprehensive characterization and immune-profiling of relevant immune cells in formalin-fixed paraffin-embedded human lymphoid tissue samples. To characterize cells harboring actively transcribed virus, authors were employed an *in-situ* hybridization assay in combination with additional protein markers (multispectral RNAscope). This type of analysis can increase the dimensionality and predictive power of flow-cytometry and single-cell RNA expression analyses, thus accelerating biomarker discovery in the context of infection and vaccination.	Moysi et al.
The GAS5 regulates TCR-mediated activation and apoptosis in CD4 T cells during HIV infection through miR-21-mediated signaling, results in prolonged survival of CD4 T cells during HIV.	Nguyen et al.
The composition of circulating innate and adaptive immune cells is altered in a large group of PLHIV receiving cART for more than six months. Specifically, certain adaptive immune responses (Th17) are preserved while IFN-g responses are compromised. The study suggests that the changes in the immune cell architecture and functional immunity in treated HIV highlight associations with the HIV reservoir, thereby emphasizing the importance of early cART initiation.	Van de Wijer et al.
This study demonstrates a direct causal relationship between the presence of HIV in platelets and T-cell dysfunctions in immunological non-responders (InRs), thereby providing valuable insights for the development of a targeted platelet therapy for improving immune reconstitution in these individuals.	Zhu et al.
This study documented the elevated levels of CD4+ Tfh and CD4+ Tscm cells, as well as an abundance of memory and effector T cells in HIV-2 infected individuals. Additionally, they found increased frequencies of CXCR5+ CD8+ T cells and CD8+ Tscm cells as well as memory B cells responsible for NAb development in HIV-2 infected persons.	Ponnan et al.
This systematic review on the dynamics of CD4+ T cells in SIV and HIV infection described the mechanism of CD4+ depletion, disease progression, and the influence of early ART on restoration.	Le Hingrat et al.
This review discussed the impact of HIV infection on Tfh cells and mucosal IgA responses in the GIT, as well as the implications these effects have on gut dysbiosis and mucosal immunopathogenesis.	Onabajo and Mattapallil
In this systematic review, authors performed a pooled data-analysis comparing the transcriptome profiles of latently- and reactivated HIV-1 infected cells of 5 *in vitro* primary CD4+ T cell models of HIV-1 latency and 2 *ex vivo* studies of reactivated HIV-1 infected primary CD4+ T cells from HIV-1 infected individuals. They observed 5 differentially expressed genes FRY, GCSAM, GNLY, GPR15 and MSRB2 and natural ligands (CCL4 & CCL5) and coreceptors (CXCR6) were predominantly downregulated that co-occurred in latently- and reactivated HIV-1 infected primary CD4+ T cells	Inderbitzin et al.
This study provided the overview of host characteristics and hyper-inflammatory response in COVID-19 and HIV infection to better understand the mechanisms of T cell dysfunction. The study summarized that both HIV-1 and SARS-CoV-2 infections are associated with CD4+ T cell loss and immunodeficiency. The study suggests that a better understanding of mechanism of T cell dysfunction will contribute to the development of targeted therapy against severe COVID-19 and will help to rationally design vaccine involving T cell response for the long-term control of viral infection	Peng et al.
In this study, reviewed the current literature on the pathogenesis of HIV/HCV coinfection, the impact of HCV coinfection on HIV disease progression in the presence of ART, the effects of HIV on HCV-associated liver morbidity, and the consequences of DAA-mediated HCV cure on immune reconstitution and HIV reservoir persistence in coinfected patients. The study also found that HIV reservoirs are higher in HCV/HIV coinfected individuals than in those with HIV mono-infection, and DAA-mediated HCV cure does not reduce HIV persistence or protect against HCV reinfection.	Gobran et al.

## Author contributions

VV, CP, MV and SKS edited this topic; SG and VV wrote the original draft; MV, CP, HB and SKS reviewed and edited the final manuscript. All authors contributed to the article and approved the submitted version.
